# Physicochemical, Nutritional, Microbiological, and Sensory Qualities of Chicken Burgers Reformulated with Mediterranean Plant Ingredients and Health-Promoting Compounds

**DOI:** 10.3390/foods10092129

**Published:** 2021-09-09

**Authors:** Ambrogina Albergamo, Rossella Vadalà, Daniela Metro, Vincenzo Nava, Giovanni Bartolomeo, Rossana Rando, Antonio Macrì, Laura Messina, Roberto Gualtieri, Nadia Colombo, Sabrina Sallemi, Michelangelo Leonardi, Vincenzo Lo Turco, Giacomo Dugo, Nicola Cicero

**Affiliations:** 1 Department of Biomedical, Dental, Morphological and Functional Images Sciences (BIOMORF), University of Messina, Viale Annunziata, 98100 Messina, Italy; rosvadala@libero.it (R.V.); dmetro@unime.it (D.M.); vnava@unime.it (V.N.); rrando@unime.it (R.R.); michelangelo.leonardi@unime.it (M.L.); vloturco@unime.it (V.L.T.); dugog@unime.it (G.D.); ncicero@unime.it (N.C.); 2 Science4Life Srl, an Academic Spin-Off of University of Messina, Viale Annunziata, 98100 Messina, Italy; gbartolomeo@unime.it (G.B.); macri.anto@outlook.it (A.M.); lauramessina1985@pec.it (L.M.); 3 Avimecc Spa, C.da Fargione, Agglomerato Industriale ASI, 97015 Modica, Italy; robertogualtieri@avimecc.com (R.G.); nadiacolombo@avimecc.com (N.C.); 4 Leocata Mangimi, Via Vanella, 162, 97015 Modica, Italy; sabrinasallemi@leocatamangimi.it

**Keywords:** poultry, functional meat, chicken burger, Mediterranean diet, bioactive compounds, proximate composition, microbiological analysis, sensorial analysis, mineral analysis, FA composition

## Abstract

The quality of chicken burgers reformulated by the partial replacement of meat by Mediterranean plant ingredients and enriched with peculiar amounts of n-3 PUFAs, Mg, Fe, Se, and folic acid, was evaluated in comparison to conventional chicken burgers. Specifically, two types of burger were developed, namely the “Sicilian burger”—based on cherry tomato and rosemary—and the “Mediterranean burger”—with basil leaves and thyme essential oil—every recipe being differentially functionalized according to the nutritional requirements of consumers, such as children, pregnant women and elderly. Mediterranean ingredients were responsible for different pH, color, and cooking loss between conventional and functional burgers. Except for n-3 PUFAs resulting poorly fortified, the functionalization with Mg, Fe, Se, and vitamin B9 was successful in all products. Considering the target consumer categories, the daily consumption of the functional burger may assure an intake of Mg, Fe, and Se equal, respectively, to 37.31–59.90%, 17.76–46.81%, and 27.20–50.05%, and a cover of vitamin B9 of 31.98–48.31% of the relative population reference intakes. Fortified products kept a good microbiological quality during 5 days of refrigerated storage, and, according to the sensorial descriptive analysis and the hedonic test, they showed a higher acceptability than conventional burgers.

## 1. Introduction

A variety of reasons, including the globalization, the loss of traditional food culture, the sedentary and busy lifestyle, on the one hand, the strong awareness of the link diet-health, and the increasingly competitive food market, on the other, have converged and propelled towards the global development of functional food. Functional food has established as a convenient solution to manage certain chronic health conditions, and it has become influential in diverse scientific and regulatory branches. Since its conception in the 80s, however, the term itself has changed its meaning in relation to the country and culture, being defined and re-defined along with the benefits it led to, thus, creating confusion among health experts, policy makers, and public [1,2], and making somewhat arduous the analysis of the relative market. Based on the common consensus in considering the functional food as a natural or processed food which, being added with ingredients/components with an additional health-value, promotes the optimal health and reduces the risk of chronic diseases [3], the global market of functional food had an estimated value of USD 173.26 billion in 2019 and a projection of USD 309.00 billion by 2027, with a compound annual growth rate of 6.7% from 2021 to 2027 [4]. Region-wise, Asia-Pacific dominated in 2019, with a market share of 46.8%, followed by the United States and Europe. Western Europe, particularly, accounts for ~16% of the global revenue, showing a market with a high number of small segments, varying from country to country due to diverse food traditions and cultural heritages. In this respect, the United Kingdom holds the greatest market share (i.e., 20% of total revenues, corresponding to ~USD 7.4 billion) followed by Germany and France with 14% and 13% of total revenues (~USD 4.9 billion). Spain and Italy account for 12% and 11% of total revenues, respectively, which correspond to ~USD 3.7 billion [5]. Although consumers have a deep awareness of the link between diet and health, as well as a high interest in the nutritional and health aspects of their food choices, the lower diffusion of functional nutrition in Italy is mainly due to (i) a tangible confusion about what a functional product is, and (ii) a strong connection with the genuine and natural Mediterranean diet, inducing inevitably a certain skepticism towards the manipulated/reformulated food of doubtful safety [6].

Considering the market segmentation by product, functional dairy, bakery and cereals currently—and are expected to be during the forecast period 2021/2027—the leading type products, followed by the segment of fish, meat and eggs [4]. Meat, particularly, shows a great functionalization potential because of (i) the great versatility, which enables producers to launch a wide array of attractive, convenient, and easy-to-use products [7], and (ii) the already significant presence of important nutrients (e.g., quality proteins, some essential fat-soluble vitamins, and minerals), with high bioavailability [8,9]. Additionally, meat processing leads to the generation of many compounds beneficial to human health, such as bioactive peptides which are inactive within the sequence of the parent protein and can be released by hydrolysis during processing [10] and improves the product’s shelf-life [11]. On the other hand, however, meat suffers from a negative consumer perception associated with the intrinsic saturated fat and cholesterol, and the potential presence of salt, synthetic preservatives, such as nitrites and nitrates, and toxic compounds, such as nitrosamines polycyclic aromatic hydrocarbons, derived by peculiar processing procedures [12]. The meat industry offers essentially three possibilities for developing functional products, such as (i) the modification of the carcass composition, (ii) the manipulation of meat raw materials, and (iii) the reformulation of meat products [13]. Considering the strategy of reformulation, the product shall be tailored into a healthier form by adding “functional” ingredients (i.e., fiber, vegetable protein, MUFAs and PUFAs, antioxidants, etc.) or by reducing/removing harmful components, such as salt and chemical additives [13], and, above all, it shall benefit from a high consumer acceptance. This is a key and complex aspect to be considered, as it is inevitably affected by product-related factors (e.g., product attributes, sensory qualities, production methods) and consumer-related variables as well (e.g., psychological and cultural factors, food traditions and dietary habits) [14,15,16].

Recent literature exploring the various aspects of meat reformulation includes the works of Mora-Gallego et al. with fermented sausages [17], Lorenzo et al. with Spanish salchichón [18], Horita et al. with Brazilian frankfurters [19], Resconi et al. with cooked ham [20], Kumar et al. with poultry meat finger chips [21], Li et al. (2019) with chicken meat batters [22], among others.

The aim of this study was to develop reformulated meat products, specifically chicken burgers, that could meet the nutritional requirements of specific consumer’s categories from the Italian large-scale distribution, such as children, pregnant women and elderly, by means of the enrichment with valuable nutrients, such as polyunsaturated fatty acids, vitamins and inorganic elements. Additionally, the chicken meat was not treated with any chemical preservative and was partially replaced with ingredients peculiar to the Mediterranean diet, in an attempt to meet Italian consumers’ food habits, thus, encouraging their awareness and acceptance towards functional food. Reformulated products were tested for their physicochemical, nutritional, and sensory properties, as well as microbiological quality and shelf-life to evaluate their marketing potential.

## 2. Material and Methods

### 2.1. Food Materials

Around 5 kg of minced chicken (thigh) meat were provided by Avimecc S.p.A. (Modica, Italy), a Sicilian company specialist in the poultry sector. Powdered fortifiers (i.e., eicosapentaenoic and docosahexaenoic acids mixture [EPA + DHA, 50:50, *w*/*w*], Mg [MgCl_2_∙6H_2_O, food grade], Fe [micronized Fe_4_(P_2_O_7_)_3_, food grade], Se [Na_2_SeO_3_, food grade], and vitamin B9) were supplied by Nuova Farmaceutica Srl (Riposto, Italy). Ingredients, such as dehydrated cherry tomato flakes, dehydrated rosemary leaves, dehydrated basil leaves, were purchased from a local market. Timo essential oil was kindly provided by Maraschi and Quirici (Torino, Italy).

### 2.2. Manufacture of Base and Fortified Burgers

Meat products were developed at the laboratory of Food Chemistry of BIOMORF Department of University of Messina (Messina, Italy) thanks to the synergic collaboration among food chemists, nutritionists, and medical experts in the field of nutrition from University of Messina and food technologists from Avimecc S.p.A. For the control condition, the formula per kg of base burger was as follows: 800 g of chicken meat and 200 g of distilled water. For the formulation of the fortified burgers, the 200 g of distilled water were replaced by 200 g of a water solution containing definite amounts of fortifiers and kept stirring until used. Two types of fortified burgers, named, respectively, “Sicilian burger” and “Mediterranean burger”, were developed for specific consumer categories, i.e., 7–10 year old male/female children, pregnant women, and 60–74 years old male/female elderly, by considering the Dietary Reference Values of Nutrients and Energy for Italian population (LARN) fixed by the Italian Society of Human Nutrition (SINU) [23].

Specifically, for the Sicilian burger, the formula per kg of product consisted of 747.5 g of chicken meat, 50 g of dehydrated cherry tomato flakes, 2.5 g of dehydrated rosemary and 200 g of fortification solution. For the Mediterranean burger, the ingredients per kg of product were 747.5 g of chicken meat, 51 g of dehydrated basil leaves, and 1.5 mg of thyme essential oil, and 200 g of fortification solution. In every case, the fortification solution was prepared with amounts of fortifiers that could provide peculiar amounts of nutrients in the final product, according to the category of consumer considered (Table 1).

The obtained emulsions were shaped using a commercial burger maker to obtain patties of ~100 g (10 cm diameter, 1 cm thickness). A total of 30 fortified burgers (three fortification treatments × two recipes × five samples for each treatment/recipe combination) were manufactured, while the control condition consisted of 10 base burgers. All burger samples were singularly wrapped in plastic packaging film and stored at +4 °C until analysis.

The success of the different fortification treatments was evaluated by means of the guidelines for the control of compliance with Reg. (EU) 1169/2011, Dir. 90/496/EEC and Dir. 2002/46/EC concerning the setting of tolerances for nutrient values declared on the label, which apply to the Reg. (EC) n. 1925/2006 on the addition of vitamins and minerals and of certain other substances to foods [24].

### 2.3. Chemicals and Reagents

For proximate composition: The Kjeldahl catalyst was supplied by Carlo Erba (Milan, Italy).

For fatty acid composition: n-heptane and n-hexane (reagent grade) were, respectively, purchased from J.T. Baker (Phillipsburg, NJ, USA). Fatty acid methyl esters (FAMEs) reference standards (C4–C24) were supplied by Supelco (Bellefonte, PA, USA).

For elemental analysis: nitric acid (65% *v*/*v*) and hydrogen peroxide (30% *v*/*v*), with trace metal analysis grade, and ultrapure water, with resistivity of 10 mΩ cm, were purchased from J.T. Baker (Milan, Italy). Stock solutions of Na, Mg, K, Ca, Fe, Cu, Mn, Zn, Se, As, Cd, and Pb (1000 mg/L in 2% HNO_3_) were from Fluka (Milan, Italy).

For microbiological analysis: Plate Count Agar (PCA), Malt Extract Agar (MEA), Tryptone Bile X-Glucuronide (TBX), Lauryl Tryptose Broth, 2% Brilliant Green Lactose Bile Broth (BGBLB), Sulphite Polymyxin Sulphadiazine (SPS) agar, Violet Red Bile Glucose Agar (VRBGA), Bacillus Cereus Agar (PEMBA), Egg Yolk Emulsion, De Man, Rogosa and Sharpe (MRS) agar, Buffered Peptone Water Selenite Cystine Broth Base, Modified Semi-solid Rappaport Vassiliadis (MSRV) medium, xylosine lysine deoxycholate (XLD), and Brilliant Green Agar (BGA), triple sugar iron (TSI) agar, urea agar, L-lysine decarboxylation medium were from Oxoid (Hampshire, UK), whereas Baird-Parker Agar Base was purchased from VWR Chemicals (Leuven, Belgium). Fraser Broth Base Half Concentration, Fraser Broth, Ferric Ammonium Citrate Supplement, Agar Listeria Acc. To Ottaviani and Agosti—ALOA, ALOA Enrichment Selective Supplement were supplied by Biolife (Milano, Italy).

### 2.4. Physicochemical Properties and Proximate Composition

For physicochemical parameters, every sample was investigated in triplicate for pH, color, and cooking loss. For the proximate composition, determinations of dietary fiber, crude protein, ash, fat, and moisture were performed in triplicate according to the AOAC official protocols of analysis [25]. Refer to Supplementary Materials for further details.

### 2.5. FA Profile

Every sample was elucidated for its FA profile according to a protocol of sample preparation and analysis already reported in Costa et al. [26], with slight modifications. Every dried lipid extract obtained by the Soxhlet apparatus was recovered through the addition of 1 mL n-hexane, and 10 drops of the extract were added with 1 mL of sodium methoxylate and heated at 100 °C during 15  min. After cooling down the solution, 1 mL of boron trifluoride/methanol (14%) was added, and again temperature was raised to 100 °C for 15  min. Approximately 1 mL of hexane was added to the cool solution, along with 4 mL of a saturated sodium chloride solution. After agitation and centrifugation, the organic layer containing fatty acid methyl esters (FAMEs) was collected and injected into a gas chromatograph (GC) equipped with a split/splitless injector and a flame ionization detector (FID) (Dani Master GC1000, Dani Instrument, Milan, Italy). A Supelco Omegawax 250 (length 30 m, 0.25 mm inner diameters, 0.25 µm film thickness, Supelco, Sigma Aldrich, St. Louis, MO, USA) was employed. The following operating conditions were used: column oven temperature from 50 (hold time 2 min) to 240 °C (hold time 15 min) at 3 °C/min; injector and detector temperatures were both set at 240 °C; helium was at a linear velocity of 30 cm/s (constant), and an initial head pressure of 99.5 KPa was set. Carrier and makeup gases were He, respectively, at a constant linear velocity of 30 cm/s and 40 mL/min; H_2_, 40 mL/min; air, 400 mL/min. The injection volume was 1 μL, with a split ratio of 1:50. Data acquisition and management was performed using Clarity Chromatography Software v4.0.2 (DataApex, Prague, Czech Republic). All samples were analyzed in triplicate along with analytical blanks. FAMEs of nutritional interest were identified by direct comparison with the retention times of compounds present in the reference standard mixture. FA concentrations were calculated in terms of mg fatty acid/100 g product.

### 2.6. Inorganic Elements

A sample aliquot of 0.5 g was mineralized with 7 mL of HNO_3_ and 1 mL of H_2_O_2_ by exploiting the microwave digestion system Ethos 1 (Milestone, Bergamo, Italy). A temperature program of 0–200 °C in 10 min (step 1), and 200 °C held for 10 min (step 2), and a microwave power of 1200 W were employed. After cooling down to room temperature, the digested sample was diluted up to 25 mL with ultrapure water. A quadrupole ICP-MS iCAP Q (Thermo Scientific, Waltham, MA, USA) was employed for the analysis. It was tuned and the method of analysis was optimized to reduce spectral (polyatomic and isobaric) and non-spectral interferences that may significantly affect the multianalyte determinations, according to what was reported already in our previous works [27,28]. The screening of minerals (i.e., Na, Mg, Ca, and K), trace essential elements (i.e., Mn, Fe, Cu, and Zn), and potentially toxic elements (i.e., As, Cd and Pb) was performed according to our optimized method [29,30]. The operating parameters were incident radio frequency (rf) power 1500 W; plasma gas flow rate [argon (Ar)] 14 L/min; auxiliary gas flow rate (Ar) 0.8 L/min; carrier gas flow rate (Ar) 1.10 L/min. The instrument was operated with helium (He) as collision cell gas (4.7 mL/min) and was equipped with a spray chamber set at 2.7 °C. The injection volume and the sample introduction flow rate were equal to 200 μL and 0.93 mL/min, respectively. Spectra acquisition occurred in full scan mode (dwell time 0.5 or 0.01 s/point, based on the analyte). All samples were screened in triplicate along with analytical blanks and data acquisition occurred through Qtegra™ Intelligent Scientific Data Solution (Thermo Scientific™). For quantification, a six-point calibration curve was built up for each analyte (*r*^2^ ranging from 0.9991 [Mg] to 0.9998 [Pb]). Triplicate measurements along with analytical blanks were carried out for every sample. The ICP-MS procedures were analytically validated in terms of linearity, limit of detection (LOD) and quantification (LOQ), accuracy, intra- and interassay variability, as reported in detail in our recent study [31].

### 2.7. Vitamin B9

Vitamin B9 was determined by a microbiological microtiterplate test (VitaFast^®^ Folic acid, R-Biopharm, Darmstadt, Germany). Approximately 1 g of homogenized product was mixed with 30 mL of phosphate buffer 0.05 mol/L (pH 8.0) and extracted for 30 min in a thermostatic bath (95 °C), under gentle stirring. Subsequently, the sample was centrifuged at 8000× *g* for 5 min, and the supernatant was collected and diluted by sterile water to obtain a sample concentration within the range of the calibration curve of vitamin B9, previously built up by means of standard solutions provided by the commercial kit. For analysis, 150 µL of suitable culture medium along with 150 µL of sample (or standard) were pipetted into predefined wells of a 96-well microplate coated with *Lactobacillus rhamnosus*. The microplate was then incubated in the dark at 37 °C for 44–48 h. The growth of *L. rhamnosus* is dependent on the supply of vitamin B9 from sample (or standard), and it is measured as turbidity and compared to the relative calibration curve. The turbidity measurement was performed by microplate reader, at a wavelength of 610–630 nm. Triplicate measurements were performed for every sample.

### 2.8. Microbiological Analysis

Microbiological quality and shelf-life were assessed in triplicate immediately after the production (T0), as well as after 3 (T1) and 5 (T2) days of storage at +4 °C, of every burger. Bacteriological tests were performed by means of standard methods of isolation, identification, and enumeration, according to the ISO requirements. Refer to Supplementary Materials for further details.

### 2.9. Sensory Analysis

The effect of meat enrichment was assessed by a descriptive sensory analysis conducted both in raw and cooked products. The sensory analysis was performed on base burgers and representative fortified samples (i.e., Sicilian and Mediterranean burgers for pregnant women) according to the standards of the ISO 13299:2016 [32] for the constitution of a 11-member panel (6 females and 5 males between 27 and 60 years old). The number of panelists was relatively restricted as they shall show comparable levels of experience and sensorial sensibility to provide accuracy, sensibility and repeatability of judgment, as well as to highlight peculiar defects or strengths of the products. All the panelists from this study met these requirements, as they were selected among technicians, researchers and professors of the University of Messina with a solid experience in the food area, including the sensorial analysis, although they were not specifically trained in the evaluation of meat.

For the descriptive analysis, the panel was trained following the criteria of ISO 8586:2012 [33]. Considering the raw product, attributes were about the appearance (intended as a combination of attractiveness/pleasantness, color pleasantness, color uniformity, and general structure), the tactile sensations (i.e., tackiness and slickness), and the odor (overall) [34].

For the cooked product, attributes such as visual aspect (i.e., combination of attractiveness/pleasantness, color pleasantness, color uniformity, and general structure), flavor (overall), taste (overall and salty), and texture (i.e., juiciness, tenderness, stringiness and chewing rest, intended as amount of meat in the mouth when ready for swallowing) were considered [35]. The intensity of each attribute was measured on a linear, non-structured scale from 0 (no intensity) to 8 (high intensity).

Overall, samples stored at 4 °C between 1 and 4 days after their production in laboratory were presented singularly in white dishes before being cooked, to evaluate the attributes expected for the raw product. Then, the cooking process was carried out by pre-heating a non-stick aluminum pan (diameter = 26 cm) for 5 min, and by cooking the burger 5 min per side, thus, ensuring an internal core temperature of 80 °C. Samples were served individually in white dishes immediately after cooking.

Triplicate sensory tests were carried out in a testing room with temperature set at 21 ± 2 °C, neutral colored wall and furniture, and standard lighting conditions. The panelists performed the analysis in individual chambers and had no specific information about the experimental design.

Alongside the descriptive analysis, a hedonic analysis was also conducted. In this respect, around 30 consumers, equally divided between children (males and female 7–8 years old) and elderly (males and females 60–74 years old) were selected among panelists’ family members and friends. However, no pregnant women were found. They were asked to cook the burgers according to the same procedure described above, taste the samples during the meal, also in accompaniment with salad or bun, and fill in a first questionnaire expressing an acceptance judgment on a 1–7 hedonic “dislike–like” scale (1 = extremely unpleasant; 7 = extremely pleasant). Each consumer tested three anonymous samples, represented by the base burger and the Sicilian and Mediterranean burgers functionalized for the relative consumer category. The second questionnaire asked consumers to judge the cooking procedure and its general effect on products, as well as the liking of the different products in combination with other foods.

### 2.10. Statistical Analysis

Experimental data were expressed as mean ± standard deviation of three replicate measurements per sample. Statistical analyses were done using the SPSS package (SPSS 21.0, Chicago, IL, USA).

The effect of the fortification on the physicochemical, proximate, nutritional, sensory properties, and microbiological stability of burgers was evaluated by one-way analysis of variance (ANOVA). When fortification effects resulted to be significant, a post-hoc Tukey’s honestly significant difference (HSD) was employed to determine significant differences among the control condition and the experimental treatments. A two-tailed Student’s *t*-test for unpaired data was carried out for evaluating the effect of “Sicilian” and “Mediterranean” recipes on fortified burgers. Statistical significance was accepted at *p* ≤ 0.05 in all statistical analyses.

## 3. Results and Discussion

The present study is part of a research project aimed to develop functional meat products by fortifying the original product with health-promoting molecules. In a previous experimental phase—not reported in this study—the conventional chicken burger revealed to be a precious matrix to develop fortified products. Nonetheless, the addition of certain fortifiers (i.e., EPA + DHA and Mg) demonstrated to affect the taste and flavor of the original product, thus, generating the rejection of the functional burger by the sensory panel. For these reasons, the present study aimed to overcome this shortcoming by incorporating herbs, spices, and essential oil, which further improved not only the nutritional and technological value of investigated products but also their flavor and taste. Considering the relevance and the wide distribution of such plant ingredients in the Mediterranean basin [36,37,38,39,40,41,42], the Italian consumer should be encouraged to approach the functional meat product within the context of the Mediterranean diet. Hence, the success of targeted fortifications on the newly developed Sicilian and Mediterranean burgers, was explored not only in terms of chemical composition, but also under the technological, microbiological, and sensorial perspectives.

### 3.1. Physicochemical Properties and Proximate Composition

Basic physicochemical proprieties of control and fortified burgers are reported in Table 2.

The different fortification treatments developed in this study significantly affected the pH, color, and the cooking loss, of chicken burgers, regardless the different consumer categories chosen for the study. The measurement of pH meat may affect many characteristics of the product, including texture and shelf-life as well [43]. In fact, poultry texture depends on the gelation of myofibrillar proteins, which, in turn, are particularly sensitivities to the physico-chemical properties of surrounding environment, pH included [44]. In general, a pH range of 5.80–6.30 may assure an optimal gelation of chicken thigh muscle, and therefore, an optimal product quality [45]. Additionally, such a pH range may guarantee a good microbiological profile of the meat, as the microbial growth is notoriously encouraged at pH > 7, leading to a higher risk of spoilage and shorter shelf life as well [46].

According to the obtained results, the pH of investigated burgers would be indicative of a good product quality, as it was 6.11 in the base formula and varied from 5.34 to 6.51 in all other burgers, thus, being significantly different between the control and treatment conditions (*p* < 0.05). However, for a given recipe, the pH did not vary significantly among the different fortification formulas (Sicilian burgers: 5.34–5.69, *p* > 0.05; Mediterranean burgers: 6.28–6.51, *p* > 0.05) (Table 2). Sicilian burgers had a significantly lower pH than control and Mediterranean counterpart (*p* < 0.05), probably due to the predominance of cherry tomato flakes conferring a slightly higher acidity to the products. On the other hand, Mediterranean burger showed a higher pH than control condition. In this respect, previous literature reported that spices and herbs may be responsible for an increased pH of the burger formula [47]. Overall, obtained pH values were basically within the range of pH of chicken burgers both conventional and incorporated with various vegetable extracts [48,49].

For the color measurements, the L* value was significantly different from that of fortified burgers (77.77 vs. 63.59–72.01, *p* < 0.05). In particular, such parameter decreased according to the following order: control burger (77.77) > Sicilian burger (70.64–72.01) > Mediterranean burger (63.59–66.26).

The a* variable was significantly different among control and fortified samples (8.12 vs. 4.89–9.31, *p* < 0.05). Indeed, Sicilian products had the highest a* values (9.01–9.6, *p* > 0.05), followed by the base burgers (8.12) and Mediterranean burgers (4.89–5.26, *p* > 0.05). As for the b* values, control and functional burgers were significantly different (20.14 vs. 7.89–19.65, *p* < 0.05). Specifically, base products were characterized by the highest b* value (20.14), followed by Sicilian (18.34–19.65, *p* > 0.05) and Mediterranean burgers (7.89–9.99, *p* > 0.05). Every color parameter showed no significant differences (*p* > 0.05) among the fortification formulas of a given recipe. According to the Student’s *t*-test, L*, a* and b* of a given fortification formula significantly varied depending on the recipe employed (*p* < 0.05, Table 2). Overall, color analysis pointed out that, compared to control burgers, Sicilian burgers displayed a darker and reddish color; while Mediterranean burgers were even darker and showed a yellowish/greenish color.

Cooking loss is closely related to sensorial properties such as taste, appearance, and juiciness of the meat product [50]. During the manufacturing of comminuted meat products, the mincing process may destroy the tissue structure, so that the water holding capacity of such product results inevitably affected [51]. Hence, the control of cooking loss stability is essential to keep the proper appearance and juiciness of meat products.

The cooking loss observed in all burger samples ranged from 6.52 to 19.78%, being significantly different (*p* < 0.05) between control and functional samples. Indeed, Sicilian (6.96–7.88%, *p* > 0.05) and Mediterranean (6.52–8.09%, *p* > 0.05) burgers fortified for different consumer categories showed cooking losses basically similar to each other. However, they were significantly lower than the cooking loss observed in control burgers (19.78%), most probably due to the addition of vegetable ingredients. In this respect, a previous work focused on chicken burgers incorporated with increasing amounts of various spices, revealed that treated products lost less liquid than the control condition during cooking (9.94–16.60% vs. 17.45%) [52]. In another study, the addition of 2% oat fiber in burger meat demonstrated to reduce cooking losses by 20–40%, thus, improving the water holding capacity of the derived product [53].

The proximate composition of investigated burgers is shown in Table 2. Protein content was significantly higher in the base formula than fortification treatments (20.27% vs. 15.05–18.11%, *p* < 0.05), regardless of the recipes exploited, which showed protein levels basically similar to each other (Sicilian recipe: 15.37–17.35%; Mediterranean recipe: 15.05–18.11%, *p* > 0.05) (Table 2). Proteins were not fortified in the burgers, hence the differences highlighted between control and treatment conditions may be due to the natural variability of the meat, as well as to the minimal replacement of meat by alternative low-protein ingredients, such as cherry tomato flakes, basil, and rosemary in the fortified samples. Overall, obtained values were in line with the range of protein revealed in conventional chicken burgers and chicken burgers incorporated with vegetable ingredients [54,55].

All functional products showed significantly higher lipid contents than base burgers (7.74–11.28% vs. 5.53%, *p* < 0.05). Specifically, lipids ranged from 7.74% to 9.08% (*p* > 0.05) in Sicilian burgers, and from 10.25% to 11.28% (*p* > 0.05) in Mediterranean burgers, being in both cases similar among the consumer categories considered in this study. According to the Student’s *t*-test, lipids of a given fortification formula significantly varied between the two recipes employed (*p* < 0.05) (Table 2).

Overall, control burgers from this study showed to be less fatty than conventional chicken burgers reported in literature (7.49–9.07%) [54,55]. On the other hand, the higher lipid content characterizing treated burgers may be explained by the replacement of low-fat meat by Mediterranean ingredients contributing somewhat to increase the fat percentage, as well as the fortification of the product with the EPA + DHA mixture.

Ash was non-significantly different in all burger samples, regardless of the control/treatment condition and recipe considered in the study. Although not statistically significant, ash was slightly higher in fortified products than base burgers (1.78–2.50% vs. 1.38%, *p* > 0.05) probably due to the fortification of meat with ingredients with noticeable mineral content, and peculiar inorganic elements (i.e., Mg, Fe and Se) (Table 2). However, considering fortified samples, no significant differences were revealed among the different fortification formulas of a given recipe (*p* > 0.05), nor between Mediterranean and Sicilian burgers intended for a certain consumer category (*p* > 0.05). Ash values from this study were basically in agreement with the ash of conventional chicken burgers and enriched with alternative ingredients as well (2.05–2.21%) [54,55].

Crude fiber varied significantly between control and treated samples (0.57% vs. 1.05–2.64%, *p* < 0.05), as the dehydrated ingredients present in Sicilian and Mediterranean burgers contributed to increase the fiber in the meat product where it would otherwise be basically absent. Sicilian burgers had significantly higher dietary fiber than Mediterranean products (1.97–2.29% vs. 1.05–1.39%, *p* < 0.05). No significant differences were highlighted among the different fortification formulas developed with the Sicilian or Mediterranean recipe (*p* > 0.05). However, according to the Student’s *t*-test, the fiber of a given fortification formula varied significantly between the Sicilian and Mediterranean products, thus, highlighting that the different plant ingredients may differentially affect the fiber content of final products (*p* < 0.05) (Table 2). The increment of fiber level in chicken burgers following the fortification with vegetable ingredients has been already highlighted in literature. Carvalho and colleagues [54], for example, observed that chicken burgers added with 10% and 30% of spinach had a fiber content respectively higher than 27.5% and 83.3% when compared to the control samples.

The LARN proposed by SINU suggests an adequate intake (AI) of fiber amounting to ~17 g/die for child and a reference intake (RI) of ~30 g/die for adults. According to the results, the consumption of 100 g of functional burger may guarantee a modest fiber intake, oscillating between 3.50% (Mediterranean burger for elderly) and 13.47% (Sicilian burger for child) of the expected AI and RI (Table 6).

### 3.2. FA Profile

The levels of FAMEs of nutritional interest of control and fortified chicken burgers are reported in Table 3. All burgers showed a high content of monounsaturated FAs (MUFAs, 689.59–4877.85 mg/100 g), followed by saturated FAs (SFAs, 507.69–3878.78 mg/100 g) and polyunsaturated FAs (PUFAs, 218.76–819.46 mg/100 g). Considering individual FAs, palmitic (C16:0) and stearic (C18:0) acids were the most abundant SFA (respectively, 346.33–2462.80 and 143.37–1294.53 mg/100 g), oleic acid (C18:1n-9) was the predominant MUFA (628.92–4584.19 mg/100 g), while linolenic acid (C18:3 n-6) stood out among PUFAs of all samples (194.68–1601.38 mg/100 g) (Table 3). Due to the lower fat content (Table 2), control burgers showed significantly lower levels of SFAs, MUFAs, PUFAs, and individual FAs as well, than functional samples (*p* < 0.05). Next up, Mediterranean products were characterized by higher levels of SFAs, MUFAs, and PUFAs than Sicilian samples. Accordingly, single FAs of a given fortification formula significantly varied according to the recipe employed (*p* < 0.05, Table 3). Significant differences (*p* < 0.05) were observed for EPA (C20:5 n-3) and DHA (C22:6 n-3)—equal to 1.02 and 0.77 mg/100 g in control samples and to 4.57–7.23 and 30.41–53.87 mg/100 g in fortified samples—and for the n-6/n-3 ratio—amounting to 12.72 and 7.71–10.91, respectively, in control and functional products (*p* < 0.05, Table 3). Based on published literature, the adequate and achievable dietary n-6/n-3 ratio is around 6:1 and should not exceed 10:1 to avoid adverse health consequences [56,57]. Except for control samples, functional burgers had a n-6/n-3 ratio <10. In particular, products designed with the various Sicilian formulas (7.71–9.10, *p* > 0.05), or with the Mediterranean pregnancy formula (7.81) may guarantee a n-6/n-3 ratio close to that recommended (Table 3).

Overall, obtained results were indicative of the fact that the addition of Mediterranean ingredients in meat, along with the EPA + DHA mixture, remarkably altered lipids of chicken burger, and its FA composition as well. Nonetheless, the poor fortification of EPA + DHA, underlined by the variation in defects of such n-3 PUFAs with respect to the expected amount (Table 1 and Table 3), may be explained by the process of oxidation normally affecting such FAs once they are incorporated in meat. In this respect, previous studies dealing with the re-formulation of various meat products (e.g., turkey/pork burgers, beef burgers, chicken surimi, Cinta Sienese burgers) with n-3 PUFA-rich ingredients (e.g., algal, fish and flaxseed oils), have already reported various strategies to preserve lipids from degradation—such as microencapsulation of PUFA-rich oils, or use of additives such as chelators/reductants/free radical scavengers [58,59,60,61,62]. Further experiments will aim to improve the stability of PUFAs in fortified poultry by exploring alternative solutions.

Regardless of the fortification formula, EPA + DHA should be equal to 100 mg in 100 g of burger (Table 1), so that the daily consumption of the product may cover the ~40% of the AI equal to 250 mg/die set by SINU for all the consumer categories. Although such a percentage cover was not achieved in the investigated samples, the consumption of a functional burger still accounted for 14.51–29.05% of the AIs fixed for children, pregnant women and elderly (Table 6).

### 3.3. Element Profile

The element profile of control and functional samples is shown in Table 4. Among minerals, Na was the most abundant element (889.70–1122.91 mg/100 g, *p* < 0.05), followed by K (415.74–643.11 mg/100 g, *p* > 0.05), Mg (32.75–143.74 mg/100 g, *p* < 0.05), and Ca (2.59–4.22 mg/100 g, *p* > 0.05) (Table 4). In particular, Mg was significantly different between the base formula and the functional samples 32.75 mg/100 g vs. 55.97–142.74 mg/100 g (*p* < 0.05), being fortified in both Sicilian and Mediterranean products (Table 4). The functionalization of burgers with such mineral was effective, as Mg levels detected in the various products varied in defect by 22.61% (Sicilian burger for child) and 6.30% (Mediterranean burger for elderly) and in excess by 5.20–38.73% (all other burgers) with respect to the expected amounts (Table 4), thus, being well within the tolerance limits listed in Table 1.

Essential trace elements were found in the order: Fe (0.17–12.64 mg/100 g, *p* < 0.05) > Zn (0.48–0.77 mg/100 g, *p* > 0.05) > Mn (0.022–0.062 mg/100 g, *p* < 0.05) > Se (1.40–29.17 µg/100 g, *p* < 0.05). Specifically, elements, such as Fe and Se, showed to be remarkably different between control and fortified samples (Fe: 0.17 mg/100 g vs. 2.31–12.64 mg/100 g, *p* < 0.05; Se: 1.40–29.17 µg/100 g, *p* < 0.05), thus, confirming to be fortified in meat. The fortification of burgers with both trace elements was successful. In fact, Fe varied in excess by 5.71–13.99% with respect to the relative expected amounts in the various functional products, while Se differed for defect by 1.87% in Sicilian burger for child and 1.16% in Mediterranean burger for pregnancy and for excess by 11.08–24.50% in the remaining products (Table 4). Hence, both Fe and Se resulted well within the tolerance limits presented in Table 1. Additionally, for a given consumer category, no significant differences of Mg, Fe and Se (*p* > 0.05) were revealed between the Sicilian and the Mediterranean product, thus, confirming that the different plant ingredients did not significantly affect the fortification of such elements (Table 4).

The outcome of fortification with Mg and Fe is basically consistent with the literature evidence. Mg salts are typically incorporated in meat for a double purpose, namely mineral fortification and reduction of dietary sodium, although they may be responsible for the generation of a bitter taste and off-flavors in the final product [63]. However, the addition of herbs and spices or of a blend of calcium, magnesium, and potassium salts, already proved to overcome this criticism in dry cured ham, bologna sausage and beef burgers [64,65,66], and as described in Section 3.6, was also effective in masking the negative impact of Mg salt on the sensory profile of Mediterranean and Sicilian chicken burgers.

Among the various forms of Fe (micronized or encapsulated), ferric pyrophosphate has been recently preferred, as it produces negligible color change nor causes sensory changes in food [63]. Such fortifier was already employed in pate meat, assuring moreover a good Fe bioavailability in rats and iron-deficient women [67,68], and, according to the experimental data from this study, it may be considered a promising fortifier also for chicken burgers.

Concerning Se, literature reported mainly studies on the meat fortification via the modification of animal feed, such as rabbit, pork, and poultry [69,70,71]. Only Garcia-Iñiguez-de-Ciriano and co-workers [72] evaluated the direct incorporation of Se-enriched yeast in a dry fermented sausage formulation, and developed a final product providing 100% of the American RDA established for Se. However, to the best knowledge of the authors, no previous case studies were reported on the direct fortification of chicken burgers by means of the inorganic salt.

As already shown in Table 1, the fortifier elements should be present in functional burgers in peculiar amounts depending on the consumer category. Specifically, considering the consumption of the burger within a healthy and balanced diet, Mg (intended as naturally present form + added form) should cover >20% and >33% of the Population Reference Intakes (PRIs) set by LARN, respectively, for children, and pregnant women/elderly; Fe (naturally present form + added form) should account for >15%, >40% and >35% of the PRIs fixed, respectively, for children, pregnant women, and elderly; while Se (naturally present form + added form) should explain >23% and >40% of the PRIs considered, respectively, for children, pregnant women/elderly. Depending on the consumer category, the daily consumption of 100 g of Sicilian/Mediterranean burger may guarantee an intake of Mg from 37.31% to 59.90% of the established AI, and an intake of Fe and Se, respectively, equal to 17.76–46.81% and 27.20–50.05% of the relative PRIs (Table 6).

Finally, among potentially toxic trace metals, all samples were characterized by As <LOD (0.008 µg/100 g), Cd between 3.07 and 4.82 µg/100 g (*p* > 0.05) and Pb ranging from 3.01 to 3.95 µg/100 g (*p* > 0.05). Such data highlighted that control and fortified products were safe in terms of heavy metals, as they did not exceed the maximum level of Cd and Pb equal, respectively, to 5 µg/100 g and Pb 10 µg/100 g (fw), fixed by the Reg. (EC) 1881/06 for the meat (excluding offal) of bovine animals, sheep, pig, and poultry [73].

### 3.4. Vitamin B9

The level of folic acid detected in control and fortified samples is reported in Table 5. Overall, the base formula had the lowest vitamin content (9.47 μg/100 g) and was significantly different (*p* < 0.05) from Sicilian (82.36–245.88 μg/100 g) and Mediterranean (79.96–289.88 μg/100 g) burgers.

For every recipe, the fortification formulas were statistically different (*p* < 0.05), confirming that they were effectively targeted to different consumer categories. However, the type of recipe did not significantly affect the content of vitamin B9 for a given formula (*p* > 0.05), probably due to the low supply of such vitamin by the plant ingredients employed during the fortification process (Table 5).

Overall, the functionalization of burgers with folic acid proved to be effective, as it varied in defect by 9.08% (Sicilian burger for pregnant women) and in excess by 0.98–32.52% (all other burgers) with respect to the expected amounts (Table 5), thus, being well within the tolerance limits listed in Table 1.

According to the LARN, the PRIs of vitamin B9 for children, pregnant women and elderly are, respectively, of 250, 600 and 400 μg/die and, as illustrated in Table 1, the consumption of the functional chicken burger within a healthy and balanced diet, should assure an intake of such bioactive (naturally present form + added form) >22%, >43.3% and >40% of the PRIs, respectively, for children, pregnant women and elderly. Obtained data are in line with what was originally expected, as the daily consumption of a functional chicken burger may cover from 31.98% to 48.31% of the PRIs, depending on the consumer category (Table 6).

To the best knowledge of the authors, this was the first attempt to re-formulate a chicken burger with vitamin B9. In fact, few previous studies were mainly focused on the re-formulation of other types of meat products with group-B vitamins. For example, Galan and colleagues [74] developed pork sausages fortified with an amount of folic acid ensuring 100% of the American recommended daily allowance (RDA) and stressed that the fortification did not affect the textural and color properties of the final product. In another work, Riccio and coworkers [75] formulated boiled ham burgers and beef burgers with B-group vitamins to evaluate the compensation degree provided by the fortification, in view of the degradation of these molecules typically occurring during cooking. They found out that a fortification of 25 μg/g of B vitamins allowed to reach the RDA, thus, highlighting that fortification of meat products with B-group vitamins is a useful and appealing practice.

### 3.5. Microbiological Analysis

The evaluation of microbiological quality and shelf life of control and functional burgers is reported in Table S1.

Colonies of mesophilic aerobic bacteria, Enterobacteriaceae, coliforms and lactic bacteria were revealed in all samples, regardless of the control/treatment condition. After production (T0), control burgers were generally characterized by significantly lower (*p* < 0.05) counts of mesophilic bacteria (4.5 × 10^3^ CFU/g), *Enterobacteriaceae* (1.4 × 10^2^ CFU/g), coliforms (1.1 × 10^2^ CFU/g) and lactic bacteria (5.3 × 10 CFU/g) than fortified samples. Particularly, Mediterranean burgers showed a higher number of positive plates than the Sicilian ones in terms of total viable counts (1.2 × 10^4^–3.3 × 10^5^ CFU/g vs. 1.2 × 10^4^–1.4 × 10^5^ CFU/g, *p* < 0.05), *Enterobacteriaceae* (4.6 × 10^2^–2.9 × 10^3^ CFU/g vs. 5.5 × 10^2^–1.1 × 10^3^ CFU/g, *p* < 0.05), lactic bacteria (3.2 × 10^3^–7.5 × 10^4^ CFU/g vs. 5.2 × 10^2^–2.9 × 10^4^ CFU/g, *p* < 0.05), but not total coliforms (2.2 × 10^2^–4.6 × 10^2^ CFU/g vs. 7.8 × 10–3.6 × 10^2^ CFU/g, *p* < 0.05) (Table S1).

Although with moderately increased bacterial counts, the trend described above was observed also after three (T1) days of storage at +4 °C (Table S1). After five days of refrigerated storage (T2), base formulas kept showing lower contamination levels of mesophilic bacteria (9.9 × 10^3^ CFU/g), *Enterobacteriaceae* (3.7 × 10^2^ CFU/g), total coliforms (5.7 × 10^2^ CFU/g) and lactic bacteria (8.3 × 10^3^ CFU/g) than fortified products. Similarly to T0, Mediterranean samples were characterized by higher numerical counts than Sicilian products of aerobic mesophiles (5.6 × 10^4^–7.9 × 10^5^ CFU/g vs. 7.6 × 10^4^–1.6 × 10^5^ CFU/g, *p* < 0.05), *Enterobacteriaceae* (1.2 × 10^3^ –7.3 × 10^3^ CFU/g vs. 1.1 × 10^3^–7.4 × 10^2^ CFU/g, *p* < 0.05), lactic bacteria (3.3 × 10^4^–4.2 × 10^5^ CFU/g vs. 2.9 × 10^4^–4.9 × 10^4^ CFU/g, *p* < 0.05), but not coliforms (4.9 × 10^2^–6.2 × 10^2^ CFU/g vs. 4.3 × 10^2^–8.8 × 10^2^ CFU/g, *p* < 0.05) ( Table S1). Generally, at every experimental time, fortification formulas of every burger type showed statistically different contamination degrees with each other (*p* < 0.05), as well as the type of recipe significantly affected the microbial profile of a given formula (*p* < 0.05) (Table S1).

Beside discussed microorganisms, further microbiological indicators, such as yeasts, fungi, sulphate-reducing anaerobes, *L. monocytogenes*, *E. coli*, *Salmonella* spp., *S. aureus*, and *B. cereus*, were analyzed, resulting basically absent in all samples (Table S1).

The presence of pathogenic and spoilage microorganisms in poultry and derived products represents worldwide a significant concern for producers, suppliers, consumers, and regulatory agencies. Bacterial contamination of these foods is undesirable but unavoidable and depends on the bacterial level of the poultry carcasses, the hygienic practices during manipulation and the time and temperature of storage. In this respect, among the investigated parameters, aerobic mesophiles, *Enterobacteriaceae*, coliforms, and *E. coli* are indicative of (i) the initial bacterial rate of meat, (ii) sanitation conditions during processing and manipulation, and (iii) microbiological safety of the final product as well [76,77]. On the other hand, conditions such as the intensive rearing, and the high-rate processing, in which carcasses remain in proximity with each other, favor the spread of pathogens such as *Salmonella* spp. and *L. monocytogenes* [78].

Internal and literature guidelines [76,77] have suggested the following microbiological limits for chicken products: total viable counts: <10^6^ CFU/g; *Enterobacteriaceae*: <10^4^ CFU/g; total coliforms: <10^3^ CFU/g; *E. coli*: <5 × 10^2^ CFU/g; *S. aureus*: <10^2^ CFU/g; *L. monocytogenes* <10^2^ CFU/g. Additionally, the Reg. (CE) 2073/2005 has established that Salmonella spp. shall be equal to 0 CFU/25g of product [79].

Although results from microbiological analysis suggest that the employment of natural antimicrobial ingredients (i.e., herbs/spices and essential oil) during the fortification process did not improve the hygienic profile of chicken burgers contrary to what was reported by other authors [80,81], based on the guideline values, all functional burgers showed still a good microbiological quality and were suitable for consumption during a five day storage period (+4 °C).

### 3.6. Sensory Analysis

**Figure 1:**
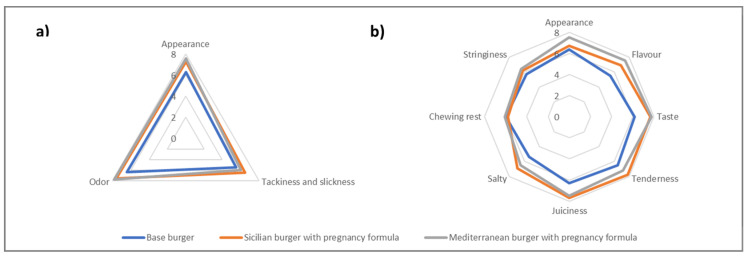
Sensory analysis of control and fortified (pregnancy woman) Sicilian and Mediterranean burgers, considering both raw (**a**) and cooked (**b**) products. Data are reported in terms of mean ± standard deviation (*n* = 11). Scores are based on a 0–8-point numeric scale.

The sensory analysis of control and representative Sicilian and Mediterranean burgers, considering both the raw and cooked products, is illustrated by the spider diagrams in Figure 1.

Results of panel test suggested that the raw/cooked base burger showed significantly lower scores for most of the attributes investigated than the raw/cooked fortified products (*p* < 0.05), regardless of the recipe involved. Considering raw products, the most significant differences were observed for the appearance (6.31 vs. 7.34–7.67, *p* < 0.05) and the odor (6.44 vs. 7.61–7.82, *p* < 0.05), as the Mediterranean ingredients gave functional products a more appealing and colorful appearance and a more pleasant spicy odor (Figure 1). Regarding cooked samples, the Mediterranean ingredients, such as tomato flakes, rosemary, basil, and thyme essential oil, were responsible for a better appearance (6.46 vs. 6.92–7.53, *p* < 0.05), flavor (5.50 vs. 7.09–7.55), and taste (6.26 vs. 7.73 and 7.86) with respect to the base formula (Figure 1). In fact, the panelists reported in general a firmer, livelier, and juicier appearance. In the mouth, the tender taste of roasted chicken well matched with the salty cherry tomato flakes and the aromatic rosemary of the Sicilian product or with the herbaceous/bitter thyme and the sweet/fresh basil of the Mediterranean recipe, without being overwhelmed. At the same time, notes such as tenderness (6.51 vs. 7.28–7.80, *p* < 0.05) and juiciness (6.37 vs. 7.56–7.72, *p* < 0.05) were significantly improved in the functional products, confirming what was visually perceived (Figure 1). In fact, plant ingredients may have improved the cooking loss of the comminuted meat product, thus, resulting in an enhanced water holding capacity [51]. Differently from what was discussed so far, attributes such as chewing rest and stringiness were not statically different between control and functional samples due probably to intrinsic characteristics of chicken meat (e.g., percentage of fat in the meat), and according to the obtained scores (6.05 vs. 5.86–6.11, *p* > 0.05), were defined as acceptable (Figure 1).

Overall, no off-odors, -flavors or -colors deriving from the presence in meat of peculiar fortifiers, such as EPA + DHA and Mg, were observed both in raw and cooked functional products, which, based on the results obtained, showed a higher degree of acceptance with respect to the conventional chicken burger. This is in line with the literature, reporting the incorporation of various ingredients—such as herbs or spices—in chicken burgers as a valid strategy not only to improve certain textural properties of meat, but also to enhance the sensorial profile of the product itself [82,83].

Results from the hedonic analysis conducted by selecting common consumers are reported in Table 7. Overall, both children and elderly showed a good liking of all products, which on average obtained scores greater than 6. Children demonstrated to equally appreciate the base and the Sicilian burgers (respectively, 6.7 and 6.3, *p* > 0.05). This was due to the fact that some of them reported to find quite “annoying” the cherry flakes and prefer simpler recipes, such as the base product and the Sicilian burger, which obtained the highest liking score (8.2, *p* < 0.05). On the other hand, elderly appreciated both functional burgers more than the base formulation (7.6–8.3 vs. 5.3, *p* < 0.05), being also in this case the Mediterranean burger the preferred product. 

For the cooking procedure, all consumers find it suitable for the type of product. A good part of them, in particular, reported that functional burgers held the cooking process better than conventional burgers. This, moreover, is in line with the findings of the descriptive analysis and of the physicochemical analysis about cooking loss and water holding capacity.

Concerning the accompaniment of the dish, most children reported to choose fries or buns; while most of elderly salad or boiled vegetables. However, all consumers reported that base and functional products were well matched with these other foods and equally appreciated.

Although a small number of participants was involved in the hedonic test, the knowledge of consumer preferences was certainly important for investigating the consumer acceptance of functional chicken burgers.

## 4. Conclusions

There is a general increasing trend in employing functional compounds during the manufacturing of meat products, due to the significant effects such compounds may exert on human health. Nonetheless, the fortification process, intended as choice of the fortifier type and quantity, and of the processing method to be done as well, may affect not only the effective outcome of the final product, but also its general acceptability. Equally important in the design of functional meat products is the consideration of the deficiencies of specific nutrients in various consumer categories, as well as their dietary habits. In the present study, the development of functional Sicilian and Mediterranean burgers occurred by considering all these variables. Although the fortification process demonstrated to be successful and the general acceptability of these products was high, certain aspects need to further be investigated, such as the improvement of the stability of PUFAs in the burger, and the nutritional/sensorial variations occurring during the shelf-life of the product and after its cooking as well.

## Figures and Tables

**Table 1:** Amounts of fortifying nutrients expected per 100 g of chicken burger intended for consumers such as children, pregnant women and elderly. Relative tolerance limits established by the guidance document for the control of compliance with Regulation (EU) No 1169/2011 are also reported. EPA: eicosapentaenoic acid; DHA: docosahexaenoic acid.

Consumer	EPA + DHA (mg)		Mg (mg)	Fe (mg)	Se (µg)	Vitamin B9 (µg)
**Child**	100		30	2	8	55
**Pregnant woman**	100		80	11	25	260
**Elderly**	100		80	3.5	22	160
	**Tolerance Limits**					
**By excess**	On total PUFAs	+0.8g	+45%			+50%
**By defect**		−0.8g	−35%			−35%

**Table 2:** Physicochemical properties and proximate composition (on a fw basis) of control and fortified Sicilian and Mediterranean burgers. Data are reported in terms of mean ± standard deviation of *n =* 10 samples for control and *n =* 5 samples for each treatment/recipe combination, where each sample was analyzed three times.

		Base Formula	Child Formula	Pregnancy Formula	Elderly Formula	Child Formula	Pregnancy Formula	Elderly Formula
			*Sicilian burger*			*Mediterranean burger*		
pH		6.11 ± 0.11 ^a^	5.69 ± 0.15 ^b^*	5.48 ± 0.13 ^b^*	5.34 ± 0.27 ^b^*	6.51 ± 0.30 ^a^*	6.28 ± 0.23 ^a^*	6.41 ± 0.07 ^a^*
Color	L*	77.77 ± 1.92 ^a^	71.83 ± 1.81 ^b^*	70.64 ± 0.56 ^b^*	72.01 ± 1.56 ^b^*	63.59 ± 2.03 ^c^*	66.26 ± 1.11 ^c^*	65.19 ± 1.39 ^c^*
	a*	8.12 ± 0.38 ^a^	9.31 ± 0.80 ^a^*	9.69 ± 0.46 ^a^	9.01 ± 0.24 ^a^*	5.09 ± 0.87 ^a^*	5.26 ± 0.98 ^a^	4.89 ± 1.55 ^a^*
	b*	20.14 ± 1.23 ^a^	19.65 ± 0.98 ^a^*	18.34 ± 1.27 ^a^*	18.89 ± 0.69 ^a^*	8.11 ± 0.75 ^b^*	7.89 ± 1.12 ^b^*	9.99 ± 0.48 ^b^*
Cooking loss (%)		19.78 ± 3.18 ^a^	6.96 ± 1.56 ^b^	7.88 ± 1.61 ^b^	7.51 ± 1.22 ^b^	8.09 ± 3.11 ^b^	7.21 ± 2.90 ^b^	6.52 ± 2.11 ^b^
Protein (%)		20.27 ± 2.67 ^a^	15.37 ± 1.10 ^b^	17.79 ± 1.34 ^b^	17.35 ± 2.23 ^b^	15.05 ± 2.30 ^b^	18.11 ± 1.13 ^b^	16.90 ± 2.09 ^b^
Lipids (%)		5.53 ± 0.12 ^a^	8.07 ± 0.30 ^b^*	7.74 ± 0.62 ^b^*	9.08 ± 1.01 ^b^*	11.28 ± 0.79 ^c^*	10.25 ± 0.90 ^c^*	10.53 ± 0.73 ^c^*
Ash (%)		1.38 ± 0.65 ^a^	2.50 ± 0.78 ^a^	2.10 ± 0.36 ^a^	1.78 ± 0.43 ^a^	2.01 ± 0.29 ^a^	1.96 ± 0.32 ^a^	2.35 ± 0.41 ^a^
Crude fiber (%)		0.57 ± 0.29 ^a^	2.29 ± 0.58 ^b^*	1.97 ± 0.22 ^b^	2.64 ± 0.52 ^b^*	1.39 ± 0.31 ^c^*	1.58 ± 0.41 ^c^	1.05 ± 0.10 ^c^*
Moisture (%)		70.20 ± 3.65 ^a^	65.12 ± 5.45 ^a^	68.36 ± 2.89 ^a^	68.09 ± 4.32 ^a^	66.37 ± 2.96 ^a^	65.24 ± 3.14 ^a^	64.97 ± 3.69 ^a^

For control and treated burgers: different superscript letters in the same row indicate significantly different values for a given parameter (*p* < 0.05 by post hoc Tukey’s honestly significant difference [HSD] test); same superscript letters in the same row indicate not significantly different values for a given parameter (*p* > 0.05 by post hoc Tukey’s HSD test). For a given fortification formula: * indicates significantly different values between *Sicilian* and *Mediterranean burgers* (*p* < 0.05 by Student’s *t*-test).

**Table 3:** Levels (mg/100 g product, fw) of individual fatty acids (FAs), saturated fatty acids (SFAs), monounsaturated fatty acids (MUFAs), and polyunsaturated fatty acid (PUFAs) of control and fortified Sicilian and Mediterranean burgers. The FAs subject to fortification (EPA + DHA) are reported in bold and italic along with the relative variation (%) from the expected fortification amount. Data are reported in terms of mean ± standard deviation of *n =* 10 samples for control and *n =* 5 samples for each treatment/recipe combination, where each sample was analyzed three times.

FA	Base Formula	Child Formula	Pregnancy Formula	Elderly Formula	Child Formula	Pregnancy Formula	Elderly Formula
		*Sicilian burger*			*Mediterranean burger*		
C14:0	16.36 ± 0.24 ^a^	111.05 ± 1.03 ^b^*	112.28 ± 1.56 ^b^*	134.08 ± 1.04 ^c^*	100.39 ± 1.39 ^d^*	89.18 ± 0.40 ^e^*	111.23 ± 1.52 ^b^*
C16:0	346.33 ± 1.37 ^a^	1651.25 ± 10.82 ^b^*	1594.35 ± 17.15 ^c^*	1949.78 ± 10.73 ^d^*	2462.80 ± 16.27 ^e^*	2329.48 ± 18.34 ^f^*	2270.25 ± 21.26 ^g^*
C18:0	143.37 ± 1.43 ^a^	795.64 ± 8.03 ^b^*	675.73 ± 13.82 ^c^*	761.87 ± 8.61 ^d^*	1294.53 ± 11.16 ^e^*	1282.62 ± 12.32 ^f^*	1040.74 ± 15.41 ^g^*
C20:0	1.64 ± 0.00 ^a^	10.76 ± 0.12 ^b^*	14.71 ± 0.24 ^c^*	17.86 ± 0.31 ^d^*	21.06 ± 0.33 ^e^*	19.13 ± 0.30 ^f^*	21.05 ± 0.26 ^e^*
SFA	**507.69 ± 2.75 ^a^**	2568.70 ± 23.48 ^b^*	2397.08 ± 27.78 ^c^*	2863.59 ± 20.55 ^d^*	3878.78 ± 20.2 ^e^*	3720.41 ± 26.06 ^f^*	3443.27 ± 26.40 ^g^*
C16:1	60.67 ± 0.74 ^a^	228.29 ± 1.26 ^b^*	215.47 ± 2.47 ^c^*	218.53 ± 3.50 ^c^*	293.66 ± 3.91 ^d^*	280.17 ± 3.92 ^e^*	239.66 ± 3.15 ^f^*
C18:1 n-9	628.92 ± 1.06 ^a^	3371.60 ± 30.91 ^b^*	3248.59 ± 28.08 ^c^*	3802.70 ± 20.68 ^d^*	4584.19 ± 29.71 ^e^*	4109.23 ± 36.45 ^f^*	4332.78 ± 27.85 ^g^*
MUFA	**689.59 ± 2.19 ^a^**	3599.88 ± 27.69 ^b^*	3464.06 ± 24.52 ^c^*	4021.23 ± 39.73 ^d^*	4877.85 ± 31.35 ^e^*	4389.39 ± 22.54 ^f^*	4572.43 ± 30.84 ^g^*
C18:2 n-6 cis	194.68 ± 1.84 ^a^	998.65 ± 11.50 ^b^*	967.92 ± 22.12 ^b^*	1146.50 ± 13.03 ^c^	1601.38 ± 18.20 ^d^*	1401.18 ± 14.16 ^e^*	1407.77 ± 7.85 ^e^*
C18:3 n-6	1.69 ± 0.00 ^a^	11.29 ± 0.33 ^b^*	8.26 ± 0.22 ^c^*	13.62 ± 0.41 ^d^*	12.41 ± 0.20 ^e^*	11.62 ± 0.13 ^b^*	11.58 ± 0.19 ^b^*
C18:3 n-3	14.41 ± 0.18 ^a^	61.31 ± 0.66 ^b^*	74.85 ± 1.87 ^c^*	77.79 ± 1.14 ^c^*	113.93 ± 1.98 ^d^*	103.87 ± 1.69 ^e^*	109.13 ± 1.64 ^f^*
C20:2 n-6	5.57 ± 0.05 ^a^	38.18 ± 0.64 ^b^*	36.14 ± 1.26 ^b^*	49.33 ± 1.02 ^c^*	45.87 ± 0.83 ^d^*	36.22 ± 0.39 ^e^*	69.48 ± 1.00 ^f^*
C20:4 n-6	0.61 ± 0.00 ^a^	4.57 ± 0.08 ^b^*	7.23 ± 0.30 ^c^*	6.96 ± 0.36 ^c^*	7.14 ± 0.17 ^c^*	5.81 ± 0.05 ^d^*	5.76 ± 0.07 ^d^*
C20:5 n-3	1.02 ± 0.05 ^a^	41.68 ± 1.03 ^b^*	43.36 ± 1.30 ^b^*	53.87 ± 1.72 ^c^*	32.71 ± 0.29 ^d^*	30.41 ± 0.36 ^e^*	32.28 ± 0.33 ^d^*
C22:6 n-3	0.77 ± 0.03 ^a^	12.64 ± 0.43 ^b^*	13.94 ± 0.51 ^b^*	18.77 ± 0.75 ^c^*	6.02 ± 0.23 ^d^*	5.87 ± 0.08 ^d^*	4.21 ± 0.07 ^e^*
*EPA + DHA*	***1.79 ± 0.04 ^a^***	54.32 ± 2.98 ^b^*	57.30 ± 3.64 ^b^*	72.64 ± 1.32 ^b^*	38.73 ± 0.59 ^c^*	36.28 ± 1.04 ^d^*	36.49 ± 0.68 ^e^*
*Variation from the expected amount*	***-***	***−47.47%***	***−44.50%***	***−29.15%***	***−63.06%***	***−65.51%***	***−65.3***
PUFA	**218.76 ± 2.66 ^a^**	1168.32 ± 13.55 ^b^*	1151.69 ± 22.69 ^b^*	1366.84 ± 14.92 ^c^*	1819.46 ± 18.23 ^d^*	1594.90 ± 14.46 ^e^*	1639.70 ± 15.30 ^e^*
n-6/n-3	**12.72 ± 1.37 ^a^**	9.10 ± 0.56 ^b^*	**7.71 ± 0.84 ^b^**	8.08 ± 0.40 ^b^*	10.91 ± 0.67 ^c^*	**7.81 ± 0.47 ^b^**	10.26 ± 0.69 ^c^*

For control and treated burgers: different superscript letters in the same row indicate significantly different values for a given parameter (*p* < 0.05 by post hoc Tukey’s HSD test); the same superscript letters in the same row indicate not significantly different values for a given parameter (*p* > 0.05 by post hoc Tukey’s HSD test). For a given fortification formula: * indicates significantly different values between *Sicilian* and *Mediterranean burgers* (*p* < 0.05 by Student’s *t*-test).

**Table 4:** Content of Na, Mg, K Ca, Fe, Zn, Mn (mg/100 g product, fw), Se, As, Cd, and Pb (μg/100 g product, fw) elements revealed in control and Sicilian and Mediterranean chicken burgers. The elements subject to fortification (Mg, Fe and Se) are reported in bold and italic along with the relative variation (%) from the expected fortification amounts. Data are reported in terms of mean ± standard deviation of *n =* 10 samples for control and of *n =* 5 samples for each treatment/recipe combination, where each sample was analyzed three times. Limit of detection (LOD) of As = 0.008 µg/100 g.

Element	Base Formula	Child Formula	Pregnancy Formula	Elderly Formula	Child Formula	Pregnancy Formula	Elderly Formula
		*Sicilian burger*			*Mediterranean burger*		
Na	1099.23 ± 58.45 ^a^	1005.58 ± 24.57 ^b^	1122.91 ± 95.11 ^a,b^	1100.28 ± 154.6 ^a,b^	889.70 ± 71.44 ^c^	950.19 ± 69.72 ^b,c^	1068.07 ± 83.71 ^b,c^
*Mg*	***32.75 ± 10.91 ^a^***	***55.97 ± 4.10 ^b^***	***139.31 ± 12.67 ^c^***	***143.74 ± 18.61 ^c^***	***68.80 ± 8.44 ^b^***	***116.86 ± 17.90 ^c^***	***107.77 ± 13.71 ^c^***
*Variation from the expected amount*	***-***	***−22.61%***	***+33.20%***	***38.73%***	***+20.16%***	***+5.13%***	***−6.30%***
K	471.18 ± 62.29 ^a,b^	415.74 ± 47.92 ^a^	558.74 ± 75.23 ^a,b^	643.11 ± 34.07 ^b^	440.24 ± 83.76 ^a,b^	551.20 ± 63.49 ^a,b^	545.09 ± 50.04 ^a,b^
Ca	2.72 ± 0.61 ^a^	2.59 ± 0.41 ^a^	2.79 ± 1.07 ^a^	3.12 ± 0.77 ^a^	3.43 ± 0.56 ^a^	2.94 ± 0.36 ^a^	4.22 ± 1.73 ^a^
*Fe*	***0.17 ± 0.05 ^a^***	***2.31 ± 0.34 ^b^***	***12.45 ± 1.33 ^c^***	***3.87 ± 0.50^d^***	***2.45 ± 0.13 ^b^***	***12.64 ± 0.97 ^c^***	***3.95 ± 0.17^d^***
*Variation from the expected amount*	***-***	***+7.00%***	***+11.63%***	***+5.71%***	***+13.99%***	***+13.36%***	***+7.99%***
Zn	0.74 ± 0.08 ^a^	0.55 ± 0.14 ^a^	0.63 ± 0.10 ^a^	0.77 ± 0.08 ^a^	0.55 ± 0.09 ^a,b^	0.75 ± 0.23 ^a^	0.48 ± 0.09 ^a,b^
Mn	0.022 ± 0.004 ^a^*	0.062 ± 0.005 ^b^*	0.035 ± 0.010 ^a^	0.029 ± 0.009 ^a^	0.033 ± 0.009 ^b^*	0.038 ± 0.009 ^b^	0.035 ± 0.012 ^b^
*Se*	***1.40 ± 0.51 ^a^***	***9.25 ± 0.60 ^b^***	***29.17 ± 4.38 ^c^***	***22.73 ± 8.78 ^c^***	***11.36 ± 4.15 ^b^***	***26.11 ± 5.93 ^c^***	***27.53 ± 2.48 ^c^***
*Variation from the expected amount*	***-***	***−1.87%***	***+11.08%***	***−3.05%***	***+24.50%***	***−1.16%***	***+18.77%***
As	<LOD	<LOD	<LOD	<LOD	<LOD	<LOD	<LOD
Cd	4.82 ± 0.57 ^a^	4.12 ± 0.66 ^a^	3.92 ± 0.36 ^a^	3.88 ± 0.24 ^a^	3.07 ± 0.73 ^a^	3.87 ± 0.18 ^a^	3.25 ± 0.66 ^a^
Pb	3.95 ± 0.18 ^a^	3.25 ± 0.78 ^a^	3.38 ± 0.66 ^a^	3.47 ± 0.52 ^a^	3.36 ± 0.14 ^a^	3.82 ± 0.40 ^a^	3.01 ± 0.39 ^a^

For control and treated burgers: different superscript letters in the same row indicate significantly different values for a given parameter (*p* < 0.05 by post hoc Tukey’s HSD test); the same superscript letters in the same row indicate not significantly different values for a given parameter (*p* > 0.05 by post hoc Tukey’s HSD test). For a given fortification formula: * indicates significantly different values between *Sicilian* and *Mediterranean burgers* (*p* < 0.05 by Student’s *t*-test).

**Table 5:** Vitamin B9 (μg/100 g, fw) detected control and Sicilian and Mediterranean chicken burgers. The variation (%) from the expected fortification amounts are reported in bold and italic. Data are reported in terms of mean ± standard deviation of *n =* 10 samples for control and of *n =* 5 samples for each treatment/recipe combination, where each sample was analyzed three times.

Analyte	Base Formula	Child Formula	Pregnancy Formula	Elderly Formula
Vitamin B9	9.47 ± 2.81 ^a^	*Sicilian burger*		
		82.36 ± 9.85 ^b^	245.88 ± 33.93 ^c^	192.37 ± 16.76 ^d*^
		***+32.52%***	***−9.08%***	***+14.34%***
		*Mediterranean burger*		
		79.96 ± 10.23 ^b^	289.88 ± 37.43 ^c^	171.04 ± 25.83 ^d*^
		***+28.16%***	***+7.85%***	***+0.98%***

For control and treated burgers: different superscript letters in the same row indicate significantly different values for a given parameter (*p* < 0.05 by post hoc Tukey’s HSD test); the same superscript letters in the same row indicate not significantly different values for a given parameter (*p* > 0.05 by post hoc Tukey’s HSD test). For a given fortification formula: * indicates significantly different values between *Sicilian* and *Mediterranean burgers* (*p* < 0.05 by Student’s *t*-test).

**Table 6:** Evaluation of the coverage of adequate intake (AI), reference intake (RI) or population reference intake (PRI) of fortifying nutrients derived from the daily consumption of Sicilian and Mediterranean burgers (100 g) by various consumer categories.

Nutrient	AI, RI or PRI *	Child	Pregnant Woman	Elderly	Child	Pregnant Woman	Elderly
		*Sicilian burger*			*Mediterranean burger*		
		*COVERAGE*					
Dietary fiber	-Male/female child (7–10 y/o): AI 2 g/MJ (or ~17g/die) -All other consumer categories: RI 3–4g/MJ (or ~30g/die)	13.47%	6.56%	8.8%	8.17%	5.26%	3.50%
EPA + DHA	-All consumer categories: AI 250 mg/die	21.73%	22.92%	29.05%	15.50%	14.51%	14.60%
Mg	-Male/female child (7–10 y/o): PRI 150 mg/die -Pregnant woman: PRI 240 mg/die -Male/female elderly (60–74 y/o): PRI 240 mg/die	37.31%	58.04%	59.90%	45.87%	48.70%	44.90
Fe	-Male/female child (7–10 y/o): PRI 13 mg/die -Pregnant woman: PRI 27 mg/die -Male/female elderly (60–74 y/o): PRI 10 mg/die	17.76%	46.11%	38.7%	18.84%	46.81%	39.5%
Se	-Male/female child (7–10 y/o): PRI 34 µg/die -Pregnant woman: PRI 60 µg/die -Male/female elderly (60–74 y/o): PRI 55 µg/die	27.20%	48.62%	41.32%	33.41%	43.52%	50.05%
Vitamin B9	-Male/female child (7–10 y/o): PRI 250 µg/die -Pregnant woman: PRI 600 µg/die -Male/female elderly (60–74 y/o): PRI 400 µg/die	32.94%	40.98%	48.09%	31.98%	48.31%	42.76%

* Values were retrieved by LARN fixed by SINU [23].

**Table 7:** Hedonic analysis involving common children and elderly consumers conducted on base and functional Sicilian and Mediterranean burgers. For the analysis *n* = 12 children and *n* = 15 elderly were recruited.

Product	Consumer Categories	
	Child (*n* = 12)	Elderly (*n* = 15)
Base burger	6.7 ± 0.8 ^a^	5.3 ± 0.7 ^a^
Sicilian burger	6.3 ± 1.7 ^a^	7.6 ± 0.4 ^b^
Mediterranean burger	8.2 ± 0.7 ^b^	8.3 ± 0.5 ^c^

Different superscript letters in the same column indicate significantly different values (*p* < 0.05 by post hoc Tukeyʼs HSD test); the same superscript letters in the same column indicate not significantly different values (*p* > 0.05 by post hoc Tukey’s HSD test).

## Data Availability

The datasets generated for this study are available on request to the corresponding author.

## Supplementary Materials

The following are available online at https://www.mdpi.com/article/10.3390/foods10092129/s1, Paragraph: Physicochemical properties and proximate composition; Paragraph: microbiological analysis; Table S1. Microbiological quality (T0) and shelf-life at three (T1) and five (T2) days of storage at +4 °C of control and fortified burgers. Data are reported in terms of mean ± standard deviation of *n =* 10 samples for the control condition, and *n =* 5 samples for each treatment/recipe combination, where each sample was analyzed three times.
