# Supplementary material for: Physicochemical, Nutritional, Microbiological, and Sensory Qualities of Chicken Burgers Reformulated with Mediterranean Plant Ingredients and Health-Promoting Compounds
Source: Foods. 2021 Sep 9;10(9):2129. doi: 10.3390/foods10092129 (PMC8471101; doi:10.3390/foods10092129)
Supplement: Supplementary file 1 [file foods-10-02129-s001.zip › foods-1356336-supplementary.pdf]

Supplementary Materials

Physicochemical properties and proximate composition

For physicochemical parameters, every sample was investigated for pH, color, and cooking loss. The pH of the burgers was measured using a digital portable pH-meter (3510, Jenway, United Kingdom). Color was measured using a portable colorimeter (CR-400, Minolta, Tokyo, Japan) with pulsed xenon arc lam, 0◦ viewing angle geometry and 8 mm aperture size, to estimate burger color in the CIELAB space: lightness (L*); redness (a*); yellowness (b*). The color was measured in three different points of each sample in homogeneous and representative areas. Cooking loss (%) was measured by the difference between weight of a raw burger and weight of the same burger after cooking. In this respect, every burger was cooked placing vacuum package bags in a thermostatic water bath until a core temperature of 70 °C was reached. Then, samples were cooled, and the cooking loss percentage was calculated [1]. Every determination occurred in triplicate.

For the proximate composition, determinations of dietary fiber, crude protein, ash, fat, and moisture were performed according to the AOAC official protocols of analysis [2].

Dietary fiber was determined following the AOAC Official Method 991.43 and AACC Method 32-07.01. For every sample, fiber was measured in duplicate by using the Megazyme assay kit (International Ireland Ltd., Wicklow, Ireland). Briefly, two sample aliquots (1.0 g each) were in parallel gelatinized at 80 °C by α-amylase and then, digested at 60 °C with protease and amyloglucosidase. The solutions were cooled at ~40 °C and treated with 4 vol. of ethanol to precipitate fiber and remove depolymerized proteins and D-glucose (from starch). The derived residues were filtered, washed (in sequence, with ethanol (78%, v/v), ethanol (95%, v/v), and acetone), dried and the mean weight was calculated. At this stage, a residue was incubated at 500 °C until constant weight (~12 h) for determining ash, whereas the other residue was analyzed for crude protein. Crude protein was determined using the AOAC Official Method 976.05. Briefly, the residue was digested with sulfuric acid (98%), copper (II) selenite dihydrate, and potassium sulfate by the

SpeedDigester K-439 (Büchi, Switzerland) and then analyzed by the KjelMaster System K-375 (Büchi, Switzerland) equipped with a scrubber of gases and vapors (Scrubber K-415, Büchi, Switzerland). The resulting solution was treated with sodium hydroxide (40%), to develop ammonia, which was subsequently distilled and collected in 50 mL of boric acid (1%). Hence, the amount of nitrogen was determined by titration with hydrogen chloride (0.1 N). For the calculation of the %protein, the obtained %nitrogen was multiplied by a conversion factor of 6.5.

Crude fiber was then calculated as the mean weight of the dried residue less the weight of protein and ash. The AOAC method 960.39 was used for total fat determination. Briefly, an aliquot (15.0 g) of each sample was extracted with n-heptane for 6 h, by a Soxhlet apparatus. Then, the total lipid extract was evaporated to dryness with a rotating evaporator, model P/N Hei-VAP Precision ML/G3 (Heidolph Instruments GmbH & Co., Schwabach, Germany), and the extraction yield was determined gravimetrically. Moisture was determined from sample weight loss after oven drying at 110 °C for 4 h (AOAC method 925.09). All sample measurements were conducted in triplicate.

Microbiological analysis

Microbiological quality and shelf-life of burgers were assessed immediately after the production (T0), as well as after 3 (T1) and 5 (T2) days of storage at +4 °C. Bacteriological tests were performed by means of standard methods of isolation, identification and enumeration, according to the ISO requirements [3-12].

A meat sample (25 g) was removed aseptically using a scalpel and forceps from every burger, transferred to a stomacher bag (BagMixer, Model CC, Interscience, Saint Nom, France), and homogenized with 225 ml Peptone-NaCl solution (0.1 + 0.8%) for 2 min at room temperature. For microbial enumeration, aliquots (1 mL) of appropriate serial dilutions were prepared and spread on the surface of dry media. Total viable counts (TVC) were determined using PCA, after incubation for 3 days at 30 °C. *Enterobacteriaceae* were counted in VRBGA, by incubation for 24 h at 37 °C. Yeasts and fungi were counted on MEA turned to pH=3.5 with lactic acid solution 10%, after incubation for

5 days at 30°C. The detection and enumeration of total coliforms occurred by the three-tube Most Probable Number (MPN). Specifically, sample aliquots were placed in tubes containing Lauryl Tryptose Broth and incubated at 37 °C for 48h, then they were examined for turbidity and gas, and the growth pattern was evaluated against a table of values. Positive readings from the tubes (i.e., turbidity and gas formation) were performed as confirmatory tests for coliforms in BGBLB, at 37 °C for 48 h. After the incubation period, the results were based on the proportion of tubes with turbidity and gas production for three consecutive dilutions and were expressed in MPN/g, then converted to colony forming unit (CFU) per g of product.

Escherichia coli enumeration was determined by inoculation on TBX, at 44 °C for 24 h. The detection of *Staphylococcus aureus* was performed on Baird Parker Agar Base, added with the addition of egg supplements yolk tellurite emulsion, followed by incubation for 24 h at 37 °C, whereas *Bacillus cereus* was determined by inoculation on PEMBA and incubation at 37°C for 24 h. For the detection of sulphate-reducing anaerobes, the inoculation was performed on a plate of SPS agar and by incubation at 37 °C for 48 h in anaerobic conditions. To evaluate the number of mesophilic lactic bacteria, the inoculation was performed in MRS agar, followed by incubation for 72 h at 30°C. For the analysis of *Salmonella* spp., the inoculation occurred into a MSRV medium, and plates were incubated for 24 h at 41.5 °C (negative samples were re-incubated for an additional 24 h). One microliter of the presumptive Salmonella growth (detected by the halo generated in MSRV after 24 or 48 h) was transferred to two selective media XLD and BGA. Suspected colonies were confirmed biochemically (i.e., TSI agar, urea agar, L-lysine decarboxylation medium, and indole reaction). The protocol for the enumeration of *L. monocytogenes* consists of a 24-h enrichment in half Fraser broth followed by a secondary enrichment in full Fraser broth for 24 h with streaking on selective ALOA-plates and another selective medium of choice for 48 h after both enrichment steps. Afterwards, suspect colonies were tested with biochemical reactions of confirmation. Every microbiological test was conducted on every sample in triplicate.

Table S1. Microbiological quality (T0) and shelf-life at three (T1) and five (T2) days of storage at +4 °C of control and fortified burgers. Data are reported in terms of mean ± standard deviation of n=10 samples for the control condition, and n=5 samples for each treatment/recipe combination, where each sample was analysed three times.

T	Parameter	Base formula	Child formula	Pregnancy formula	Elderly formula	Child formula	Pregnancy formula	Elderly formula
			Sicilian burger			*Mediterranean burger*		
0	**Total viable counts at 30°C** (CFU/g)	$4.5\times10^3\pm1.3\times10^{a*}$	$1.7\times10^4\pm0.7\times10^{2b*}$	$1.2\times10^4\pm1.4\times10^{2c*}$	$1.4\times10^5\pm7.2\times10^{3d*}$	$1.2\times10^4\pm3.9\times10^{2c*}$	$3.3\times10^5\pm2.1\times10^{2d*}$	$7.9\times10^2\pm8.6\times10^{e*}$
	**Enterobacteriaceae** (CFU/g)	$1.4\times10^2\pm3.9\times10^{a}$	$6.0\times10^2\pm1.7\times10^{b*}$	$5.5\times10^2\pm0.67\times10^{c*}$	$1.1\times10^3\pm1.2\times10^{2d*}$	$4.6\times10^2\pm1.9\times10^{e*}$	$2.9\times10^3\pm2.1\times10^{2f*}$	$8.7\times10^2\pm6.1\times10^{g*}$
	**Yeasts** (CFU/g)	Absent	Absent	Absent	Absent	Absent	Absent	Absent
	**Fungi** (CFU/g)	Absent	Absent	Absent	Absent	Absent	Absent	Absent
	**Total coliforms** (CFU/g)	$1.1\times10^2\pm3.4\times10^{a}$	$4.6\times10^2\pm5.1\times10^{b*}$	$2.2\times10^2\pm9.1\times10^{c*}$	$4.3\times10^2\pm7.3\times10^{b*}$	$7.8\times10\pm0.7\times10^{a*}$	$3.6\times10^2\pm0.3\times10^{d*}$	$9.8\times10\pm0.7\times10^{e*}$
	E. Coli (CFU/g)	Absent	Absent	Absent	Absent	Absent	Absent	Absent
	S. aureus (CFU/g)	Absent	Absent	Absent	Absent	Absent	Absent	Absent
	B. cereus (CFU/g)	Absent	Absent	Absent	Absent	Absent	Absent	Absent
	**Sulphate-reducing anaerobes** (CFU/g)	Absent	Absent	Absent	Absent	Absent	Absent	Absent
	**Lactic bacteria** (CFU/g)	$5.3\times10\pm1.2\times10^{a*}$	$4.8\times10^3\pm1.6\times10^{2b*}$	$5.2\times10^2\pm15\times10^{c*}$	$2.9\times10^4\pm3.1\times10^{2d*}$	$3.2\times10^3\pm9.6\times10^{2e*}$	$2.7\times10^4\pm1.0\times10^{2d*}$	$7.5\times10^4\pm3.8\times10^{2f*}$
	L. monocytogenes (CFU/g)	Absent	Absent	Absent	Absent	Absent	Absent	Absent
	***Salmonella* spp.** (CFU/25g)	Absent	Absent	Absent	Absent	Absent	Absent	Absent
			Sicilian burger			*Mediterranean burger*		
1	**Total viable counts at 30°C** (CFU/g)	$6.9\times10^3\pm2.7\times10^{2a*}$	$2.9\times10^4\pm5.6\times10^{3b*}$	$2.7\times10^4\pm6.1\times10^{3b*}$	$2.9\times10^5\pm3.3\times10^{4c*}$	$3.1\times10^4\pm6.7\times10^{2d*}$	$5.7\times10^5\pm3.3\times10^{2e*}$	$4.7\times10^3\pm5.4\times10^{f*}$
	**Enterobacteriaceae** (CFU/g)	$1.9\times10^2\pm5.1\times10^{a*}$	$6.8\times10^2\pm1.4\times10^{b*}$	$6.2\times10^2\pm5.7\times10^{b*}$	$6.8\times10^2\pm2.5\times10^{b*}$	$5.1\times10^2\pm2.8\times10^{c*}$	$6.8\times10^3\pm1.5\times10^{2d*}$	$2.1\times10^3\pm2.2\times10^{ec*}$
	**Yeasts** (CFU/g)	Absent	Absent	Absent	Absent	Absent	Absent	Absent

	**Fungi** (CFU/g)	Absent	Absent	Absent	Absent	Absent	Absent	Absent
	**Total coliforms** (CFU/g)	$2.3\times10^{2}\pm1.7\times10^{a}$	$6.8\times10^{2}\pm3.9\times10^{b*}$	$2.5\times10^{2}\pm1.3\times10^{a*}$	$5.8\times10^{2}\pm8.8\times10^{b*}$	$2.7\times10^{2}\pm9.6\times10^{a*}$	$4.2\times10^{2}\pm4.3\times10^{c*}$	$1.3\times10^{2}\pm2.7\times10^{d*}$
	E. Coli (CFU/g)	Absent	Absent	Absent	Absent	Absent	Absent	Absent
	S. aureus (CFU/g)	Absent	Absent	Absent	Absent	Absent	Absent	Absent
	B. cereus (CFU/g)	Absent	Absent	Absent	Absent	Absent	Absent	Absent
	**Sulphate-reducing anaerobes** (CFU/g)	Absent	Absent	Absent	Absent	Absent	Absent	Absent
	**Lactic bacteria** (CFU/g)	$9.3\times10^{2}\pm4.3\times10^{a}$	$2.0\times10^{4}\pm2.7\times10^{3b*}$	$2.1\times10^{4}\pm2.0\times10^{3b*}$	$1.1\times10^{4}\pm6.5\times10^{3c*}$	$2.9\times10^{4}\pm4.2\times10^{2d*}$	$9.7\times10^{4}\pm3.9\times10^{2e*}$	$1.3\times10^{5}\pm2.2\times10^{3f*}$
	L. monocytogenes (CFU/g)	Absent	Absent	Absent	Absent	Absent	Absent	Absent
	***Salmonella* spp.** (CFU/25g)	Absent	Absent	Absent	Absent	Absent	Absent	Absent
			Sicilian burger			*Mediterranean burger*		
	**Total viable counts at 30°C** (CFU/g)	$9.9\times10^{3}\pm5.1\times10^{2a}$	$1.4\times10^{5}\pm2.7\times10^{2b*}$	$7.6\times10^{4}\pm3.5\times10^{2c*}$	$1.6\times10^{5}\pm3.7\times10^{4d*}$	$4.9\times10^{5}\pm6.7\times10^{2e*}$	$7.9\times10^{5}\pm1.0\times10^{2f*}$	$5.6\times10^{4}\pm7.2\times10^{2g*}$
	**Enterobacteriaceae** (CFU/g)	$3.7\times10^{2}\pm1.9\times10^{a}$	$1.1\times10^{3}\pm4.1\times10^{2b}$	$7.4\times10^{2}\pm3.3\times10^{c*}$	$1.3\times10^{3}\pm4.2\times10^{2b*}$	$1.2\times10^{3}\pm1.5\times10^{2b}$	$7.3\times10^{3}\pm1.7\times10^{2d*}$	$4.9\times10^{3}\pm3.8\times10^{2d*}$
	**Yeasts** (CFU/g)	Absent	Absent	Absent	Absent	Absent	Absent	Absent
	**Fungi** (CFU/g)	Absent	Absent	Absent	Absent	Absent	Absent	Absent
	**Total coliforms** (CFU/g)	$5.7\times10^{2}\pm2.3\times10^{a}$	$8.8\times10^{2}\pm1.9\times10^{b*}$	$4.3\times10^{2}\pm2.5\times10^{c*}$	$7.9\times10^{2}\pm1.5\times10^{d*}$	$4.9\times10^{2}\pm2.5\times10^{2e*}$	$6.2\times10^{2}\pm2.7\times10^{f*}$	$5.4\times10^{2}\pm0.9\times10^{g*}$
**2**	***E. Coli*** (CFU/g)	Absent	Absent	Absent	Absent	Absent	Absent	Absent
	S. aureus (CFU/g)	Absent	Absent	Absent	Absent	Absent	Absent	Absent
	B. cereus (CFU/g)	Absent	Absent	Absent	Absent	Absent	Absent	Absent
	**Sulphate-reducing anaerobes** (CFU/g)	Absent	Absent	Absent	Absent	Absent	Absent	Absent
	**Lactic bacteria** (CFU/g)	$8.3\times10^{3}\pm2.6\times10^{2a}$	$2.9\times10^{4}\pm1.8\times10^{3b*}$	$4.9\times10^{4}\pm4.2\times10^{3c*}$	$3.2\times10^{4}\pm2.9\times10^{3d*}$	$3.3\times10^{4}\pm5.8\times10^{2d}$*	$2.6\times10^{5}\pm3.9\times10^{2e*}$	$4.2\times10^{5}\pm4.5\times10^{3f*}$
	L. monocytogenes (CFU/g)	Absent	Absent	Absent	Absent	Absent	Absent	Absent

	***Salmonella* spp.** (CFU/25g)	Absent	Absent	Absent	Absent	Absent	Absent	Absent

For control and treated burgers: different superscript letters in the same row indicate significantly different values for a given parameter (P < 0.05 by post hoc Tukey's HSD test); same superscript letters in the same row indicate not significantly different values for a given parameter (P > 0.05 by post hoc Tukey's HSD test). For a given fortification formula: * indicates significantly different values between Sicilian and Mediterranean burgers (P < 0.05 by Student's t-test).

Internal guideline values: Total viable counts at 30°C: $<10^6$ CFU/g; Enterobacteriaceae: $<10^4$ CFU/g; Yeasts: $<10^4$ CFU/g; Fungi: $<10^4$ CFU/g; Total coliforms: $<10^3$ CFU/g; E. Coli: $<5\times10^2$ CFU/g; S. aureus: $<10^2$ CFU/g; B. cereus: $<10^2$ CFU/g; Sulphate-reducing anaerobes $<10^2$ CFU/g; Lactic bacteria $<10^6$ CFU/g; L. monocytogenes $<10^2$ CFU/g. Guideline value from Reg. (CE) 2073/2005: Salmonella spp.: 0 CFU/25g.

References

[1] Lorenzo. J. M., Pateiro. M., & Franco. D. (2013). Influence of muscle type on physicochemical and sensory properties of foal meat. *Meat science*. *94*(1). 77-83.

[2] AOAC. (2012). AOAC official methods of analysis (18th ed.). Gaithersburg. MD. USA: Association of Official Analytical Chemists Arlington.

[3] ISO 11290-1:2017. (2017). Microbiology of the food chain — Horizontal method for the detection and enumeration of Listeria monocytogenes and of Listeria spp. — Part 1: Detection method

[4] ISO 15213:2003. (2003). Microbiology of food and animal feeding stuffs — Horizontal method for the enumeration of sulfite-reducing bacteria growing under anaerobic conditions.

[5] ISO 16649-1:2018. (2018). Microbiology of the food chain — Horizontal method for the enumeration of beta-glucuronidase-positive Escherichia coli — Part 1: Colony-count technique at 44 degrees C using membranes and 5-bromo-4-chloro-3-indolyl beta-D-glucuronide.

[6] ISO 21527-1:2008. (2008). Microbiology of food and animal feeding stuffs — Horizontal method for the enumeration of yeasts and moulds — Part 1: Colony count technique in products with water activity greater than 0.95.

[7] ISO 21528-2:2017. (2017). Microbiology of the food chain — Horizontal method for the detection and enumeration of Enterobacteriaceae — Part 2: Colony-count technique.

[8] ISO 4832:2006. (2006). Microbiology of food and animal feeding stuffs — Horizontal method for the enumeration of coliforms — Colony-count technique.

[9] ISO 4833:2003. (2013). Microbiology of food and animal feeding stuffs. Horizontal method for the enumeration of microorganisms. Colony-count technique at 30 °C.

[10] ISO 6579-1:2017. (2017). Microbiology of the food chain — Horizontal method for the detection. enumeration and serotyping of Salmonella — Part 1: Detection of Salmonella spp.

[11] ISO 6888-1. (2021). Microbiology of the food chain — Horizontal method for the enumeration of coagulase-positive staphylococci (Staphylococcus aureus and other species) — Part 1: Method using Baird-Parker agar medium.

[12] ISO 7932:2004. (2004). Microbiology of food and animal feeding stuffs — Horizontal method for the enumeration of presumptive Bacillus cereus — Colony-count technique at 30 degrees C.
